# Supplementary material for: Inhibition of hepatic lipogenesis enhances liver tumorigenesis by increasing antioxidant defence and promoting cell survival
Source: Nat Commun. 2017 Mar 14;8:14689. doi: 10.1038/ncomms14689 (PMC5424065; doi:10.1038/ncomms14689)
Supplement: Supplementary Information — Supplementary Figures [file ncomms14689-s1.pdf]

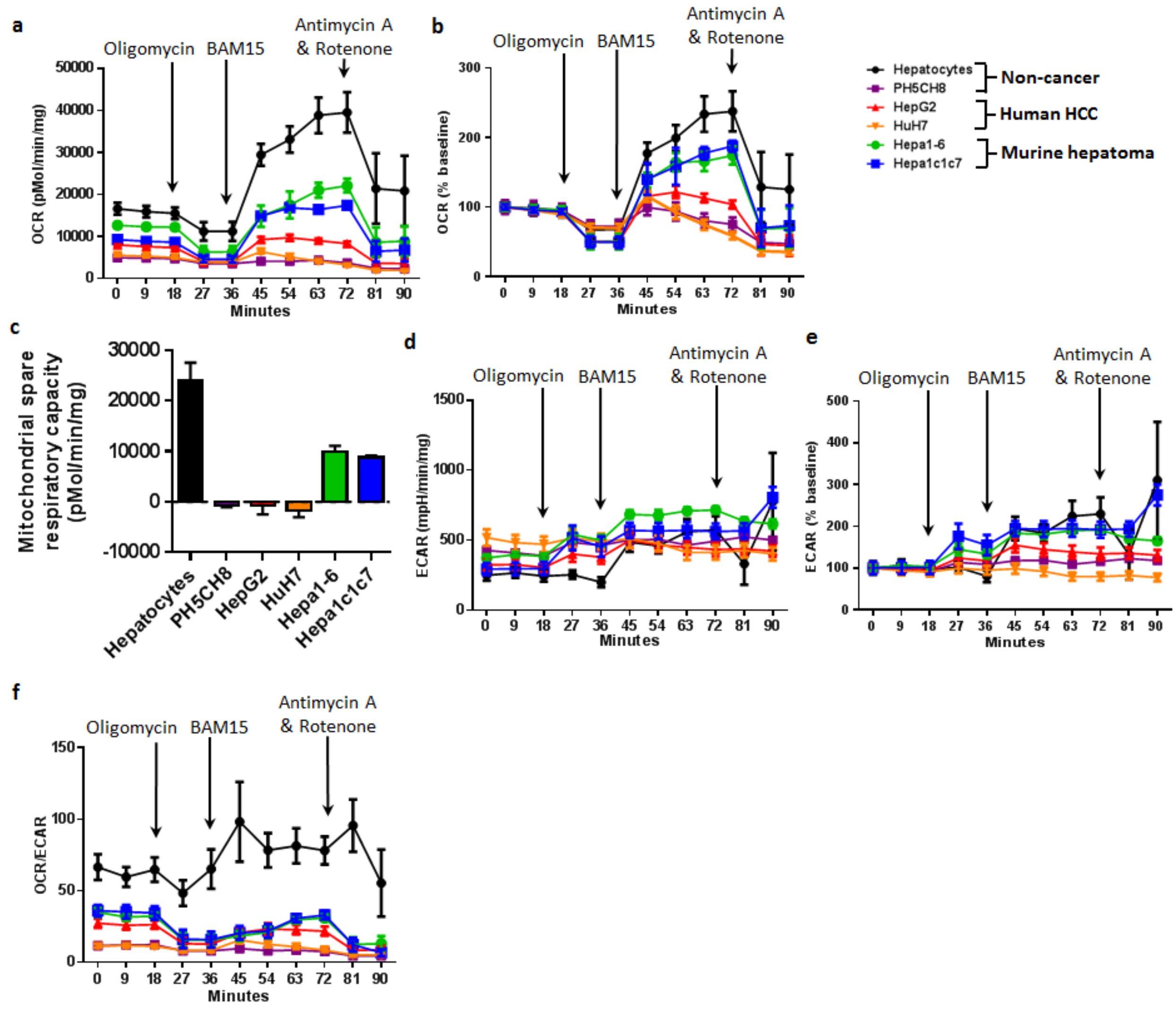

Supplementary Figure 1. Mitochondrial function in liver non-cancer and tumor cells. Mitochondrial stress test in whole cells using oligomycin (2μM), BAM15 (2μM), Antimycin A (10μM), and Rotenone (1μM) as indicated. Oxygen consumption rate (OCR) **(a)** normalized to protein or **(b)** normalized to basal OCR and **(c)** mitochondrial spare respiratory capacity in non-cancerous cells (primary murine hepatocytes and the immortalized human liver cell line PH5CH8), human liver cancer cell lines (HepG2 and Huh7), and murine liver cancer cell lines (Hepa1-6 and Hepa1c1c7). Extra-cellular acidification rate (ECAR) **(d)** normalized to protein or **(e)** normalized to basal ECAR**. (f)** Ratio of OCR to ECAR (OCR/ECAR) (n=3 independent experiments). Data are represented as mean ± SEM.

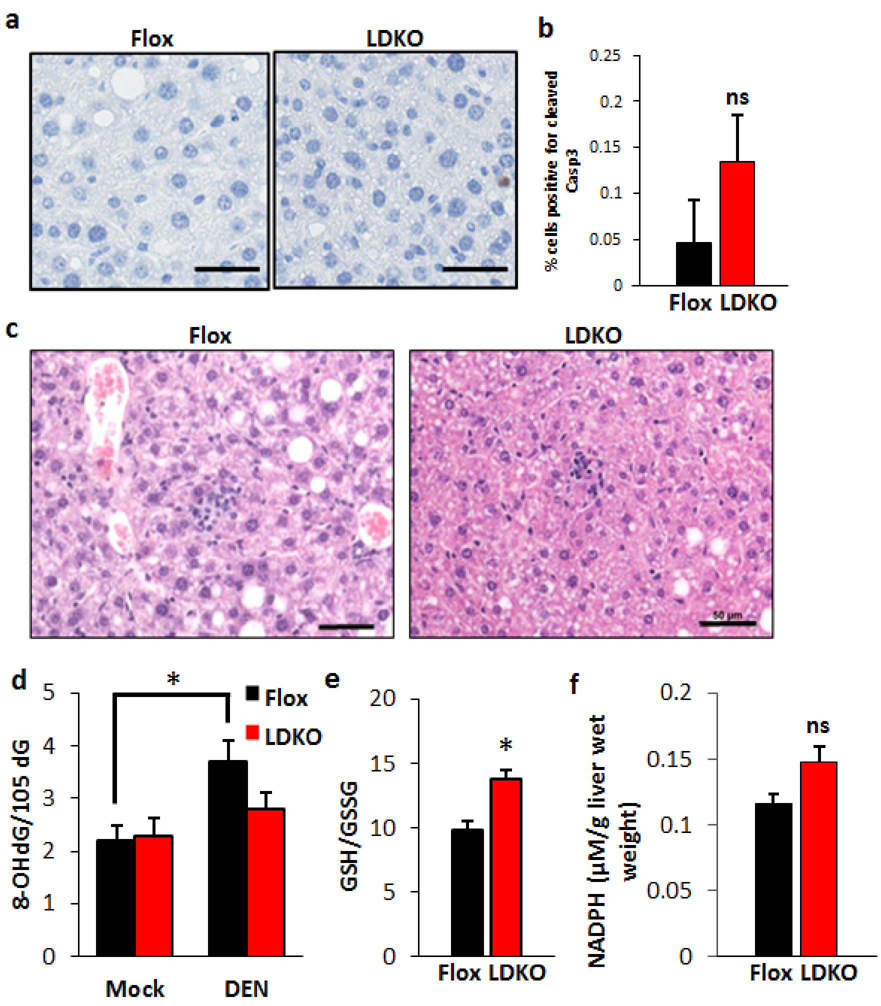

Supplementary Figure 2. Cleaved CASP3 staining, inflammation, and antioxidant status in livers of DEN-treated Flox and LDKO mice. (a) Representative images and **(b)** quantification of cleaved Casp3 immunohistochemical staining in livers of DEN-treated Flox and LDKO mice at 40w of age, scale bars=50 µm. **(c)** Representative H&E-stained livers from Flox and LDKO mice depicting inflammatory cell infiltration, scale bars = 50 µm. For **a-c**, n=10 Flox and 11 LDKO mice. **(d)** 8-OHdG levels of DNA isolated from Flox and LDKO liver tissue 24h after DEN treatment. **(e)** GSH:GSSG ratios and **(f)** NADPH levels in liver tissue of Flox and LDKO mice 24h after DEN treatment. For **d-f**, n=4 mice. * indicates significant difference, $p < 0.05$ as determined by two-tailed t-test (**d** was analyzed by one-way ANOVA followed by Tukey's post hoc analysis). Data are represented as mean ± SEM.

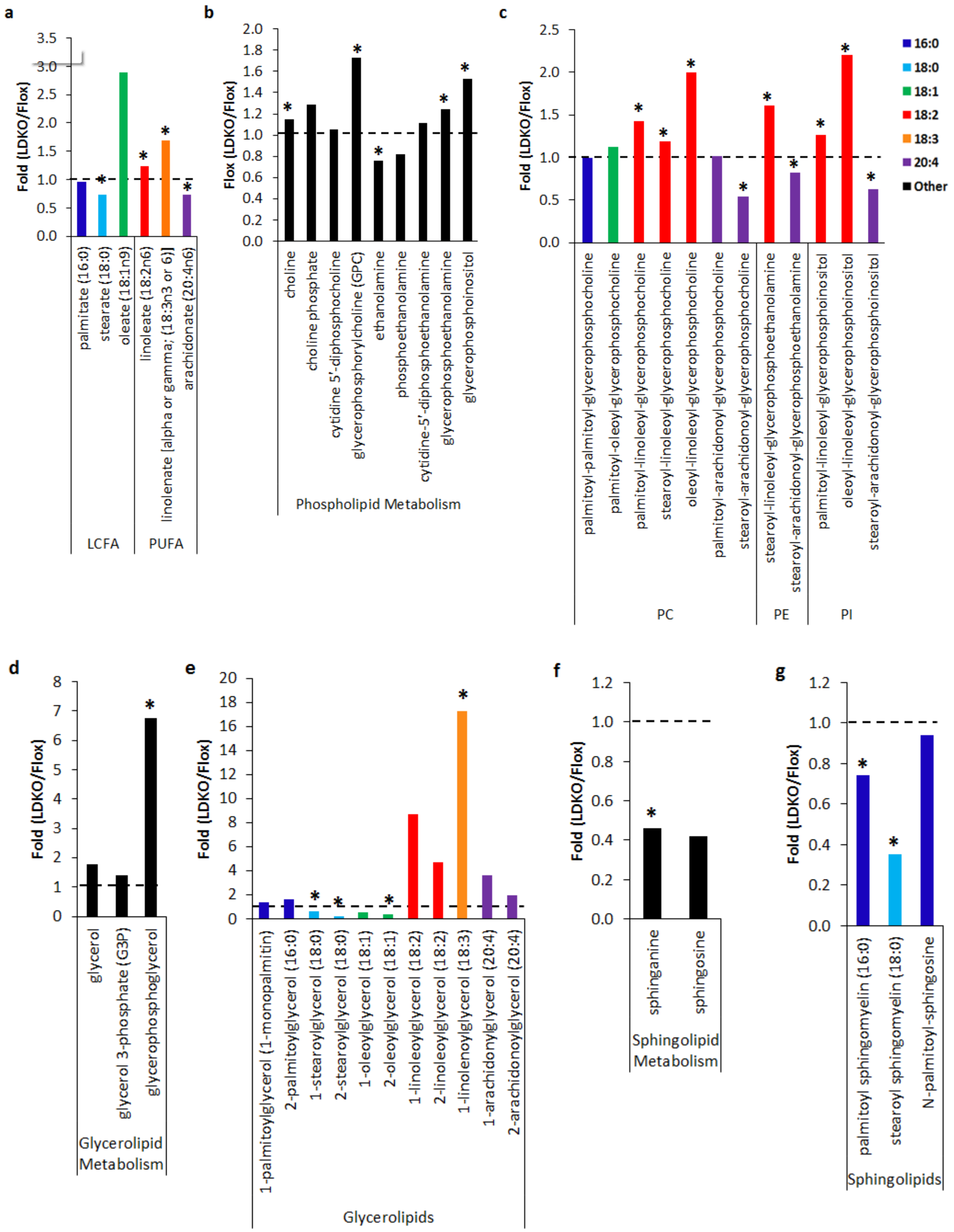
a
Fold (LDKO/Flox)
3.5
3.0
2.5
2.0
1.5
1.0
0.5
0.0
palmitate (16:0)
stearate (18:0)
oleate (18:1n9)
linoleate (18:2n6)
linolenate [alpha or gamma; (18:3n3 or 6)]
arachidonate (20:4n6)
LCFA
PUFA
b
Flox (LDKO/Flox)
2.0
1.8
1.6
1.4
1.2
1.0
0.8
0.6
0.4
0.2
0.0
choline
choline phosphate
cytidine 5'-diphosphocholine
glycerophosphorylcholine (GPC)
ethanolamine
phosphoethanolamine
cytidine-5'-diphosphoethanolamine
glycerophosphoethanolamine
glycerophosphoinositol
Phospholipid Metabolism
c
Fold (LDKO/Flox)
2.5
2.0
1.5
1.0
0.5
0.0
16:0
18:0
18:1
18:2
18:3
20:4
Other
palmitoyl-palmitoyl-glycerophosphocholine
palmitoyl-oleoyl-glycerophosphocholine
palmitoyl-linoleoyl-glycerophosphocholine
stearoyl-linoleoyl-glycerophosphocholine
oleoyl-linoleoyl-glycerophosphocholine
palmitoyl-arachidonoyl-glycerophosphocholine
stearoyl-arachidonoyl-glycerophosphocholine
stearoyl-linoleoyl-glycerophosphoethanolamine
stearoyl-arachidonoyl-glycerophosphoethanolamine
palmitoyl-linoleoyl-glycerophosphoinositol
oleoyl-linoleoyl-glycerophosphoinositol
stearoyl-arachidonoyl-glycerophosphoinositol
PC
PE
PI
d
Fold (LDKO/Flox)
8
7
6
5
4
3
2
1
0
glycerol
glycerol 3-phosphate (G3P)
glycerophosphoglycerol
Glycerolipid Metabolism
e
Fold (LDKO/Flox)
20
18
16
14
12
10
8
6
4
2
0
1-palmitoylglycerol (1-monopalmitin)
2-palmitoylglycerol (16:0)
1-stearoylglycerol (18:0)
2-stearoylglycerol (18:0)
1-oleoylglycerol (18:1)
2-oleoylglycerol (18:1)
1-linoleoylglycerol (18:2)
2-linoleoylglycerol (18:2)
1-linolenoylglycerol (18:3)
1-arachidonylglycerol (20:4)
2-arachidonoylglycerol (20:4)
Glycerolipids
f
Fold (LDKO/Flox)
1.2
1.0
0.8
0.6
0.4
0.2
0.0
sphinganine
sphingosine
Sphingolipid Metabolism
g
Fold (LDKO/Flox)
1.2
1.0
0.8
0.6
0.4
0.2
0.0
palmitoyl sphingomyelin (16:0)
stearoyl sphingomyelin (18:0)
N-palmitoyl-sphingosine
Sphingolipids

Supplementary Figure 3. Analysis of specific lipid species and lipid-related metabolites. Levels of **(a)** fatty acid species, **(b)** metabolites involved in phospholipid metabolism, **(c)** phospholipid (PC=phosphatidylcholine, PE=phosphatidylethanolamine, PI=phosphatidylinositol) species, **(d)** metabolites involved in glycerolipid metabolism, **(e)** glycerolipid species, **(f)** metabolites involved in sphingolipid metabolism and **(g)** sphingolipid species in livers of DEN-treated Flox and LDKO mice at 40w of age. * indicates significant difference from Flox, $p < 0.05$ (n=6 mice) as determined by Welch's two-tailed t-test. Data are represented as mean.

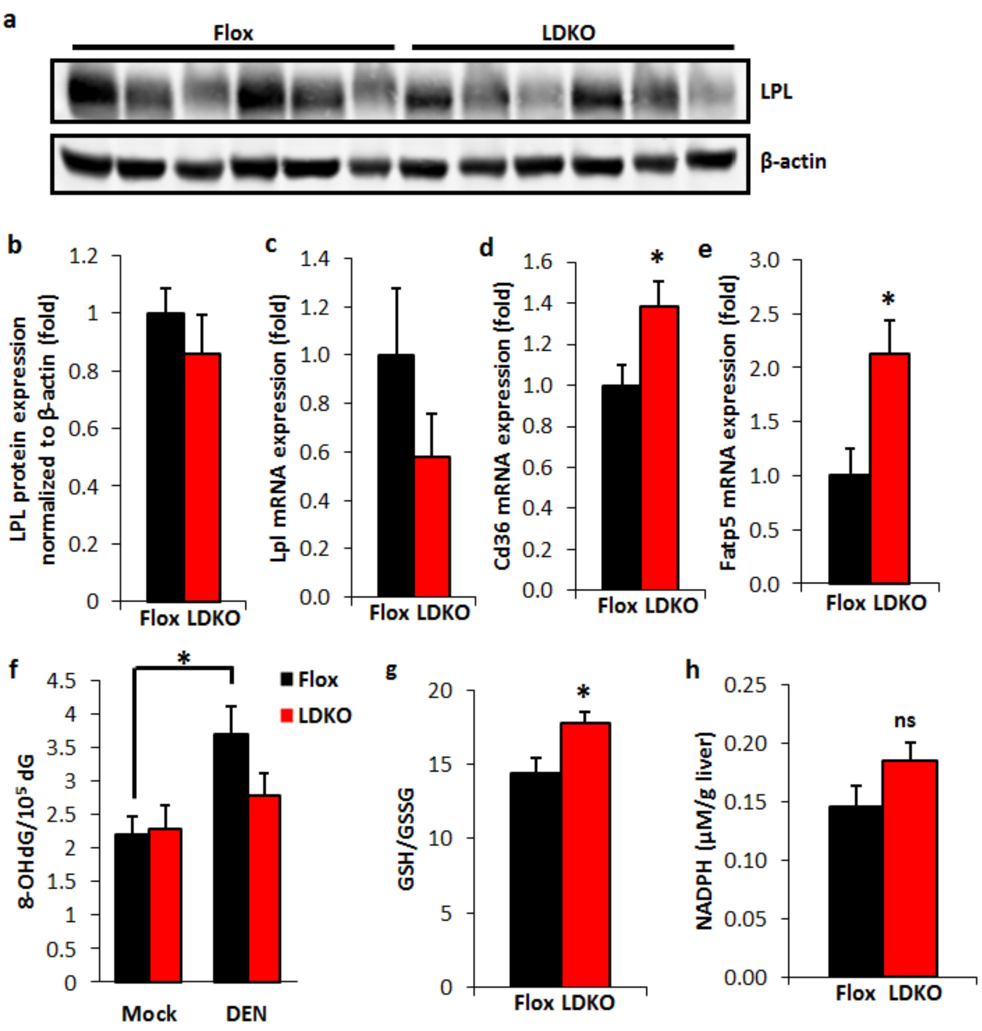

Supplementary Figure 4. Effect of ACC inhibition on lipid transporter expression and effect of acute DEN on antioxidant status. **(a)** Western blot and **(b)** quantification of LPL protein expression and mRNA expression of **(c)** *Lpl*, **(d)** *Cd36* and **(e)** *Fatp5* in livers of DEN-treated Flox and LDKO mice at 40w of age. **(f)** 8-OHdG levels of DNA isolated from Flox and LDKO liver tissue 24h after DEN treatment. **(g)** GSH:GSSG ratios and **(h)** NADPH levels in liver tissue of Flox and LDKO mice 24h after DEN treatment. * indicates significant difference, $p < 0.05$ as determined by two-tailed t-test (**f** was analyzed by one-way ANOVA followed by Tukey's post hoc analysis) n=4 mice. Data are represented as mean ± SEM.

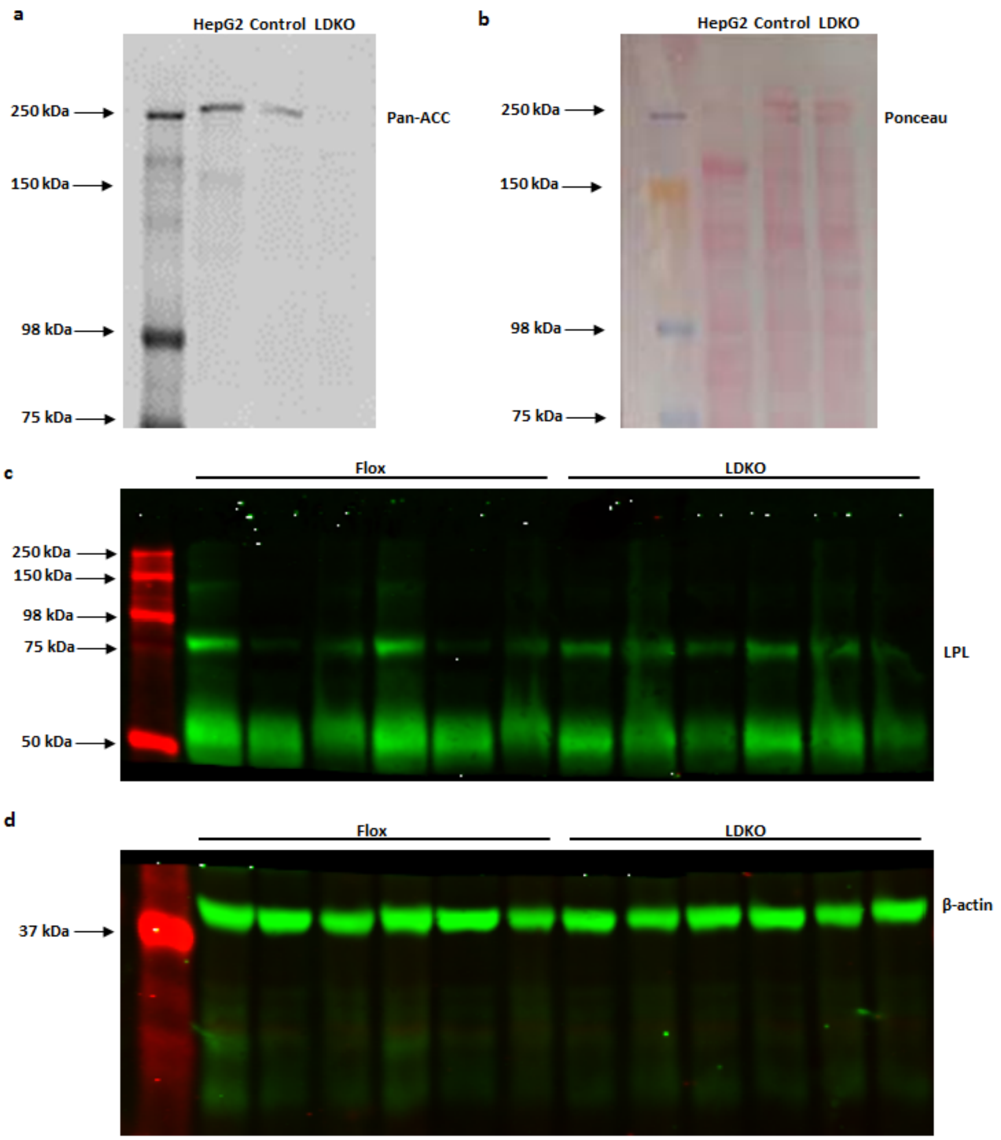

Supplementary Figure 5. Uncropped Western blots. **(a)** Western blot of pan-ACC and **(b)** ponceau stain corresponding to Figure 2d. Western blots of **(c)** LPL and **(d)** β-actin corresponding to Supplementary Figure 4a.
